# Bone Related Health Status in Adolescent Cyclists

**DOI:** 10.1371/journal.pone.0024841

**Published:** 2011-09-30

**Authors:** Hugo Olmedillas, Alejandro González-Agüero, Luís A. Moreno, José A. Casajús, Germán Vicente-Rodríguez

**Affiliations:** 1 GENUD “Growth, Exercise, Nutrition and Development” Research Group, Universidad de Zaragoza, Zaragoza, Spain; 2 Department of Psychiatry and Nursing, Faculty of Health and Sport Science (FCSD), Universidad de Zaragoza, Huesca, Spain; 3 School of Health Science (EUCS), Universidad de Zaragoza, Zaragoza, Spain; Universidad Europea de Madrid, Spain

## Abstract

**Purpose:**

To describe bone status and analyse bone mass in adolescent cyclists.

**Methods:**

Male road cyclists (n = 22) who had been training for a minimum of 2 years and a maximum of 7 years with a volume of 10 h/w, were compared to age-matched controls (n = 22) involved in recreational sports activities. Subjects were divided in 2 groups based on age: adolescents under 17 yrs (cyclists, n = 11; controls, n = 13) and over 17 yrs (cyclists, n = 11; controls, n = 9). Peak oxygen uptake (VO_2_max) was measured on a cycloergometer. Whole body, lumbar spine, and hip bone mineral content (BMC), density (BMD) and bone area were assessed using dual x-ray absorptiometry (DXA). Volumetric BMD (vBMD) and bone mineral apparent density (BMAD) were also estimated.

**Results:**

The BMC of cyclists was lower for the whole body, pelvis, femoral neck and legs; BMD for the pelvis, hip, legs and whole body and legs bone area was lower but higher in the hip area (all, P≤0.05) after adjusting by lean mass and height. The BMC of young cyclists was 10% lower in the leg and 8% higher in the hip area than young controls (P≤0.05). The BMC of cyclists over 17 yrs was 26.5%, 15.8% and 14.4% lower BMC at the pelvis, femoral neck and legs respectively while the BMD was 8.9% to 24.5% lower for the whole body, pelvis, total hip, trochanter, intertrochanter, femoral neck and legs and 17.1% lower the vBMD at the femoral neck (all P≤0.05). Grouped by age interaction was found in both pelvis and hip BMC and BMD and in femoral neck vBMD (all P≤0.05).

**Conclusion:**

Cycling performed throughout adolescence may negatively affect bone health, then compromising the acquisition of peak bone mass.

## Introduction

The role of exercise in regulating bone health is still not well understood. However healthy bone is typically related to increased mechanical loading [1]. The magnitude of the strain could prevent and treat low bone mineral density (BMD) [2], [3] or increase the acquisition of bone mass during growth [4].

Cycling can be considered a healthy sport because it improves physical fitness and prevents fat accumulation [5], [6]. Adolescence is a sensitive phase for the acquisition of bone mass [7], [8]. Around 90% of bone mass is present at the end of the skeletal maturation phase [9]. Many professional and master cyclists can be classified as osteopenic [10], [11], [12]. Professional road cyclists have significantly lower bone mineral density (BMD) than the non-active population [10]. It is assumed that this non weight-bearing activity an insufficient stimulus to generate osteogenesis in clinically relevant bone sites [12], [13].

Rico et al. [14] did not find differences in the BMC in total or any regional site between adolescent cyclists and age-matched sedentary controls. Similarly, Duncan et al. [7] observed that female adolescent cyclists had similar BMD values for the whole body, lumbar, femoral neck, legs and arms than non-athlete controls. These researchers also found no differences among groups in mid-femur for the BMC and volumetric bone mineral density (vBMD) measured by computed tomography [15]. However, when these adolescent cyclists were compared with a group of runners, female cyclists showed significant lower values for BMD for the whole body, femoral neck and legs, and lower bone strength [15].

We hypothesized that cycling during adolescence is associated with lower bone mass acquisition compared a healthy adolescent population. Therefore the main aim of this investigation was to describe the bone status in adolescent cyclists compared to a healthy age-matched group. A secondary aim was to analyse the effect of years of cycling practice on the on the acquisition of bone mass.

## Materials and Methods

### Ethics statement

Written informed consent was obtained from parents and adolescents [16]. The study was performed following the ethical guidelines of the Declaration of Helsinki 1961 (revision of Edinburgh 2000). The protocol study was approved by the Ethics Committee of Clinical Research from the Government of Aragón (CEICA; Spain).

### Subjects

Forty-four healthy male adolescents agreed to participate in the study (Table 1). To be included, subjects had to be bellow 21 years of age, healthy, without any chronic disease and free of musculoskeletal conditions, bone fractures, medications or habits affecting bone development. Twenty-two adolescent male road cyclists (CY) were recruited from different cycling teams from Aragon (Spain). All cyclists were regular participants in regional competitions, and had been participating in training sessions and competitions a mean 10 hours per week (h/week) for a minimum of 2 and a maximum of 7 years prior to the study. Twenty-two controls (CG), physically-active boys, were recruited among high school and physical education course university students. Control subjects were enrolled in recreational sports (rugby, tennis, handball, soccer) 2 h a week with occasional match at the weekend but none cycled more than 1 h per week. Cyclists and controls were divided into two subgroups, younger (<17 yr: n = 24, 11 cyclists and 13 controls) and older (>17 yr: n = 20, 11 cyclists and 9 controls), (Table 2). Subjects were asked to answer a medical and physical activity questionnaire and the parents gave additional information regarding physical activity, medical information such as past injuries, medication, known diseases and smoking habits.

**Table 1:** Subject characteristics.

	Controls (n = 22)			Cyclists (n = 22)		
	Mean		SD	Mean		SD
Age (years)	16.7	±	2.1	16.9	±	1.9
Height (cm)	176.1	±	8.9	173.2	±	6.7
Body mass (kg)	74.4	±	16.8	61.3	±	7.7*
Body fat mass (kg)	15.6	±	8.7	8.8	±	3.0*
% total body fat	21.8	±	8.5	15.7	±	4.5*
Total lean mass (kg)	56.9	±	10.7	50.7	±	6.6*
VO_2peak_ (mL kg^−1^ min^−1^)	41.7	±	9.0	56.6	±	9.3*
Years of cycling training (yr)				3.7	±	1.9
Hours of cycling training (h/sem)				10.2	±	1.8

Values are presented as mean ± SD.

*P<0.05 compared to controls. Peak oxygen uptake (VO_2_max).

**Table 2:** Subject characteristics.

	Controls						Cyclists					
	<17 yr			≥17 yr			<17 yr			≥17 yr		
	Mean		SD	Mean		SD	Mean		SD	Mean		SD
Age (years)	15.2	±	1.2	18.7	±	1.2	15.5	±	0.9	18.4	±	1.4
Height (cm)	173.3	±	9.8	180.2	±	5.7	171.1	±	5.9	175.3	±	7.0
Body mass (kg)	72.7	±	18.4	76.9	±	14.9	56.9	±	5.1$	65.6	±	7.8
Body fat mass (kg)	17	±	8.8	16	±	9.4	9.4	±	3.5$	10.1	±	2.6
% total body fat	22.9	±	8.4	20.1	±	7.5	16.6	±	5.6	15.5	±	2.2
Total lean mass (kg)	54.9	±	12.6	59.8	±	6.8	46.8	±	5.1	54.6	±	5.5
VO_2peak_ (mL kg^−1^ min^−1^)	40.2	±	9.5	43.5	±	8.6	55.2	±	11.7$	57.9	±	6.4*
Years of cycling training (yr)							2.7	±	1.3	4.4	±	1.9λ
Hours of cycling training (h/sem)							8.6	±	1.0	11.4	±	1.3λ

Values are presented as mean ± SD.

*P<0.05 compared to control group ≥17 yr.

$ P<0.05 compared to control group <17 yr.

λ P<0.05 compared to Cyclists <17 yr.

Peak oxygen uptake (VO_2_max).

### Cardiorespiratory test

Cardiorespiratory tests were performed at the same time of the day (09∶00–13∶00 h) on an electrically braked cycle-ergometer (Ergoselect 200 K, Ergoline; Bitz, Alemania). Subjects refrained from performing physical activity during the 24 h-period before the tests. After a warm-up period of 3-min with no load, power output was increased from an initial value of 30 W with 30 W increments every minute. Subjects maintained pedal cadence within the range of 60–80 rev·min^−1^. The cadence monitor was placed in view of the subject during each test and a designated investigator ensured that they maintained the required pedalling cadence throughout the duration of the test. The tests were terminated upon volitional exhaustion of the subjects and/or when cadence could not be maintained at a minimum of 60 rev·min^−1^. Peak oxygen consumption (VO_2peak_) was determined with a breath by breath gas analyzer (Oxycon Pro, Jaeger/Viasys, Germany). The gas analyzer was calibrated with a known gas prior to the first test each day, as recommended by the company. Electrocardiogram (ECG) was recorded by heart rate, utilizing a 12-lead system at rest, during the whole test and the first 3 minutes of recovery.

### Bone, lean and fat mass

Subjects were scanned in order to obtain bone measurements of the whole body, pelvis, hip, lumbar spine, head and average of arms and legs. The bone mass and lean mass [body mass – (fat mass + bone mass)] were measured using DXA (paediatric version of the software QDR-Explorer, Hologic Corp., Software version 12.4, Waltham, MA, USA). DXA equipment was calibrated using a lumbar spine phantom as recommended by the manufacturer. Subjects were scanned in supine position and the scans were performed at high resolution [17]. Lean mass (g), fat mass (g), total area (cm^2^) and BMC (g) were calculated from total and regional analysis of the whole body scan. BMD (g · cm^−2^) was calculated using the formula BMD = BMC area^−2^. The regional analysis (upper and lower extremities and pelvic region) was performed as described elsewhere [17]. Also, examinations were conducted to estimate bone mass at the lumbar spine (L1–L4) and hip regions as previously described [18]. Two additional examinations were conducted to estimate bone mass at the lumbar spine (L1–L4) and proximal region of the femur (hip and femoral neck). Volumetric BMD (vBMD) was estimated for the lumbar spine and femoral neck using simple geometric cylindrical models [19]. Bone mineral apparent density (BMAD) was calculated as previously described [20], using the formula whole body BMAD = BMC/(area^2^/body height).

### Statistics

As descriptive statistics, mean and standard deviation (SD) are given for raw data bone mass related variables and mean and standard error for bone mass adjusted results. Normality of data distribution was evaluated with Kolmogorov-Smirnov test.

To determine differences between age-groups in bone mass, one-way ANOVA with Bonferroni post hoc was applied. For adjusted results, one-way analysis of covariance (ANCOVA) with Bonferroni post hoc was used, including as covariates: body size (height) and whole body lean mass. Effect size statistics using Cohen's *d* (standardized mean difference) were calculated [21]. Taking into account the cut-offs established by Cohen, the effect size can be small (∼0.2), medium (∼0.5) or large (∼0.8). SPSS version 15.0 was used for the analysis. The probability value for the significance level was fixed at 0.05.

## Results

Table 1 and Table 2 summarize the descriptive characteristics of the participants. Cyclists and control groups were comparable in age and height. Cyclists had significantly lower body mass, total lean mass, body fat percentage, total body fat and BMI (all, P<0.01). VO_2peak_ was higher in cyclists than in controls (P<0.01).

Raw data for bone related variables is presented in Table 3. In general, lower BMC, BMD and bone area was observed in cyclists compared to controls (P<0.05, Table 3). Bone values adjusted for the combined influence of height and total lean mass are presented in table 4. Compared to controls, cyclists had lower BMC for the whole-body, pelvis, femoral neck and legs (P<0.01). BMD at the legs, pelvis, total hip and in the trochanter and inter-trochanteric subregion was lower in cyclists than in controls (P<0.01). Cyclists had lower bone area than controls at the whole-body, legs and femoral neck site (P<0.01). Total hip area was greater in cyclists than in controls (P<0.01).

**Table 3:** Bone mineral content (BMC), density (BMD) and area in control and cyclists group.

	Controls			Cyclists			
	Mean		SD	Mean		SD	*P* *
*BMC (g)*							
Whole body	2103.4	±	461.6	1665.3	±	318.3	0.001
Pelvis	312.7	±	87.1	233.1	±	48.8	0.001
Hip	41.6	±	9.3	36.6	±	6.0	0.041
Trochanter	10.2	±	2.5	8.4	±	1.7	0.007
Inter- trochanter	26.0	±	6.6	23.6	±	4.1	0.158
Femoral neck	5.3	±	0.9	4.5	±	0.7	0.030
Lumbar spine	64.4	±	16.1	53.3	±	11.4	0.011
Average arms	167.0	±	41.1	140.0	±	33.4	0.022
Average legs	535.6	±	108.3	419.5	±	77.9	0.001
Head	491.3	±	97.9	465.1	±	109.1	0.405
*BMD (g cm^−2^)*							
Whole body	1.061	±	0.115	0.933	±	0.084	0.001
Pelvis	1.243	±	0.202	0.998	±	0.115	0.001
Hip	1.072	±	0.132	0.949	±	0.088	0.001
Trochanter	0.877	±	0.095	0.741	±	0.086	0.001
Inter-trochanter	1.201	±	0.164	1.073	±	0.941	0.001
Femoral-neck	0.985	±	0.132	0.873	±	0.112	0.004
Lumbar spine	0.998	±	0.157	0.875	±	0.111	0.005
Average arms	0.762	±	0.078	0.717	±	0.617	0.001
Average legs	1.325	±	0.144	1.161	±	0.119	0.001
Head	2.030	±	0.335	1.965	±	0.383	0.553
*vBMD (g·cm^−3^)*							
Femoral neck	0.354	±	0.051	0.324	±	0.044	0.050
Lumbar spine	0.274	±	0.032	0.257	±	0.029	0.087
BMAD	0.130	±	0.171	0.091	±	0.006	0.291
*Area (cm^−2^)*							
Whole body	1963.43	±	258.89	1771.89	±	181.99	0.001
Pelvis	248.03	±	43.52	231.7	±	26.26	0.139
Hip	38.44	±	5.80	38.37	±	3.77	0.160
Trochanter	11.57	±	1.88	11.27	±	1.19	0.529
Inter-trochanter	21.52	±	4.25	21.96	±	2.80	0.688
Femoral-neck	5.35	±	0.44	5.14	±	0.37	0.091
Lumbar spine	63.90	±	9.11	60.48	±	6.58	0.959
Average arms	216.82	±	36.79	193.12	±	30.14	0.024
Average legs	401.7	±	50.29	359.31	±	33.79	0.002
Head	240.92	±	14.20	235.52	±	14.18	0.213

Values as mean and SD.

* P<0.05 compared with control group. Volumetric bone mineral density (vBMD); bone mineral apparent density (BMAD).

**Table 4:** Adjusted bone mineral content (BMC), density (BMD) and area in control.

	Controls			Cyclists			
	Mean		SD	Mean		SD	*P* *
*BMC (g)*							
Whole body	2438.1	±	39.3	2287.0	±	39.3	0.012
Pelvis	291.2	±	7.8	254.6	±	7.8	0.003
Hip	39.2	±	0.9	38.9	±	0.9	0.777
Trochanter	9.7	±	0.3	9.0	±	0.3	0.135
Inter-trochanter	24.5	±	0.7	25.2	±	0.7	0.533
Femoral neck	5.1	±	0.1	4.7	±	0.1	0.054
Lumbar spine	60.8	±	2.1	56.9	±	2.1	0.200
Average arms	154.7	±	3.2	152.3	±	3.2	0.606
Average legs	506.1	±	8.4	449.0	±	8.4	0.001
Head	464.6	±	15.7	491.9	±	15.7	0.241
*BMD (g cm^−2^)*							
Whole body	1.132	±	0.016	1.089	±	0.016	0.065
Pelvis	1.200	±	0.026	1.042	±	0.026	0.001
Hip	1.045	±	0.190	0.976	±	0.190	0.015
Trochanter	0.860	±	0.160	0.760	±	0.160	0.001
Inter-trochanter	1.170	±	0.023	1.104	±	0.023	0.054
Femoral neck	0.956	±	0.200	0.903	±	0.020	0.082
Lumbar spine	0.961	±	0.200	0.912	±	0.200	0.110
Average arms	0.742	±	0.090	0.738	±	0.090	0.787
Average legs	1.287	±	0.018	1.199	±	0.018	0.002
Head	1.940	±	0.060	2.050	±	0.060	0.214
*vBMD (g cm^−3^)*							
Femoral neck	0.347	±	0.008	0.331	±	0.008	0.160
Lumbar spine	0.268	±	0.006	0.264	±	0.006	0.579
BMAD	0.127	±	0.027	0.094	±	0.027	0.412
Area (cm^−2^)							
Whole body	2133.25	±	15.65	2078.5	±	15.65	0.021
Pelvis	238.44	±	3.97	241.29	±	3.97	0.626
Hip	37.22	±	0.54	39.59	±	0.54	0.004
Trochanter	11.50	±	0.02	11.80	±	0.02	0.115
Inter-trochanter	20.75	±	0.53	22.73	±	0.53	0.014
Femoral neck	5.30	±	0.06	5.19	±	0.06	0.249
Lumbar spine	62.46	±	1.13	61.92	±	1.13	0.746
Average arms	206.34	±	3.33	203.60	±	3.33	0.576
Average legs	390.78	±	4.98	370.22	±	4.98	0.007
Head	238.12	±	2.17	238.31	±	2.17	0.952

Values as mean and SD.

*P<0.05 compared to control group. Volumetric bone mineral density (vBMD); bone mineral apparent density (BMAD).

Table 5, 6 & 7 summarize raw data for bone based on whether cyclists and controls were younger or older than 17 years. Cyclists under 17 had lower BMC in the arms and legs and lower BMD in the whole body, pelvis, throchanter, arms and legs; and cyclists over 17 had lower BMC in the pelvis, femoral neck and legs, and lower BMD in the pelvis, hip, throchanter, femoral neck and legs (all P<0.05, Table 5, 6). Table 8, 9 & 10 present the bone values adjusted by height and total lean mass. When differences between groups were compared by age, cyclists under 17 had 10% lower BMC in the legs and 8% higher total hip area than age-matched controls (P<0.05). In groups over 17, cyclists had 26.5%, 15.8% and 14.4% lower BMC than controls at the pelvis, femoral neck and legs, respectively (P<0.05). BMD was lower in cyclists over 17 than in age-matched controls at the pelvis, hip, trochanter, inter-trochanter, femoral neck and legs (the percentages being 24.5, 10.5, 16.2, 7.5, 15.8 and 14.3, respectively, P<0.05). In addition, cyclists had a vBMD 17.1% lower than controls at the femoral neck (P<0.05, Table 9).

**Table 5:** Bone mineral content (BMC), density (BMD) and area in control and cyclist group under 17 (<17 yr) and equal or over (≥17 yr).

	Controls							Cyclists						
	<17 yr			≥17 yr			*P* $	<17 yr			≥17 yr			*P* *
*BMC (g)*	Mean		SD	Mean		SD		Mean		SD	Mean		SD	
Whole body	2469.0	±	606.5	2776.5	±	409.7	0.043	1934.7	±	285.9	2325.9	±	436.6	0.213
Pelvis	286.3	±	93.1	351.0	±	64.2	0.077	214.8	±	46.3	251.4	±	46.0	0.012
Hip	38.2	±	9.3	46.4	±	7.5	1	34.4	±	5.8	38.7	±	5.8	0.146
Trochanter	9.6	±	2.4	11.2	±	2.5	0.393	8.0	±	1.5	8.8	±	1.8	0.101
Inter-trochanter	23.6	±	7.0	29.5	±	4.4	1.000	22.1	±	4.2	25.2	±	3.5	0.400
Femoral neck	5.0	±	0.8	5.7	±	0.9	0.287	4.3	±	0.5	4.7	±	0.9	0.032
Lumbar spine	59.2	±	16.3	72.0	±	13.1	0.394	49.0	±	9.8	57.7	±	11.6	0.114
Average arms	160.2	±	47.9	176.9	±	28.4	0.048	120.2	±	24.0	159.9	±	30.8	1
Average legs	515.0	±	120.7	565.4	±	85.0	0.012	390.1	±	57.6	449.0	±	86.8	0.043
Head	468.0	±	101.3	525.2	±	87.0	1	412.7	±	76.2	517.4	±	114.8	1

Values as mean and SD. P values calculated with ANOVA.

*P<0.05 compared to control group ≥17 yr.

$ P<0.05 compared to control group <17 yr.

**Table 6:** Bone mineral density (BMD) and volumetric density (vBMD) in control and cyclist group under 17 (<17 yr) and equal or over (≥17 yr).

	Controls							Cyclists						
	<17 yr			≥17 yr			*P* $	<17 yr			≥17 yr			*P* *
*BMD (g cm^−2^)*	Mean		SD	Mean		SD		Mean		SD	Mean		SD	
Whole body	1.133	±	0.127	1.215	±	0.119	0.036	1.002	±	0.926	1.106	±	0.097	0.204
Pelvis	1.183	±	0.164	1.33	±	0.229	0.110	0.966	±	0.109	1.03	±	0.118	0.001
Hip	1.04	±	0.115	1.119	±	0.148	0.174	0.937	±	0.096	0.96	±	0.083	0.017
Trochanter	0.859	±	0.83	0.903	±	0.11	0.020	0.741	±	0.931	0.742	±	0.826	0.002
Inter-trochanter	1.166	±	0.146	1.251	±	0.185	0.290	1.055	±	0.101	1.091	±	0.087	0.063
Femoral neck	0.946	±	0.09	1.042	±	0.166	0.713	0.867	±	0.109	0.879	±	0.119	0.027
Lumbar spine	0.957	±	0.136	1.057	±	0.174	0.118	0.528	±	0.113	0.923	±	0.09	0.164
Average arms	0.753	±	0.894	0.776	±	0.062	0.076	0.753	±	0.18	0.74	±	0.073	1
Average legs	1.288	±	0.151	1.377	±	0.124	0.005	1.219	±	0.112	1.243	±	0.155	0.046
Head	1.953	±	0.346	2.141	±	0.301	1	1.788	±	0.329	2.142	±	0.363	1
*vBMD (g cm^−3^)*														
Femoral neck	0.346	±	0.036	0.364	±	0.068	1	0.334	±	0.051	0.315	±	0.036	0.171
Lumbar spine	0.27	±	0.022	0.28	±	0.044	0.268	0.244	±	0.027	0.271	±	0.026	1
BMAD	0.091	±	0.006	0.185	±	0.265	1	0.092	±	0.007	0.093	±	0.005	0.551

Values as mean and SD. P values calculated with ANOVA.

*P<0.05 compared to control group ≥17 yr.

$ P<0.05 compared to control group <17 yr.

Bone mineral apparent density (BMAD).

**Table 7:** Bone mineral area in control and cyclist group under 17 (<17 yr) and equal or over (≥17 yr).

	Controls							Cyclists						
	<17 yr			≥17 yr			*P* $	<17 yr			≥17 yr			*P* *
*Area (cm^−2^)*	Mean		SD	Mean		SD		Mean		SD	Mean		SD	
Whole body	2152.31	±	314.47	2279.52	±	176.06	0.117	1925.74	±	147.61	2089.6	±	203.38	0.418
Pelvis	236.67	±	50.27	264.45	±	25.86	1	220.31	±	26.73	243.09	±	21.16	1
Hip	36.32	±	6.09	41.52	±	3.81	1	36.58	±	3.53	40.156	±	3.21	1
Trochanter	11.08	±	2.02	12.28	±	1.46	1	10.7	±	0.95	11.83	±	1.17	1
Inter-trochanter	19.98	±	4.43	23.75	±	2.94	1	20.87	±	2.96	23.04	±	2.26	1
Femoral neck	5.26	±	0.48	5.49	±	0.39	0.742	5.01	±	0.32	5.28	±	0.39	1
Lumbar spine	60.82	±	9.42	68.35	±	6.85	1	58.83	±	5.46	62.124	±	7.43	0.449
Average arms	209.51	±	42.75	227.37	±	24.49	0.062	175.22	±	22.28	211.01	±	26.29	1
Average legs	395.88	±	57.38	410.09	±	39.55	0.115	352.58	±	30.76	366.03	±	36.77	0.175
Head	238.28	±	15.47	244.75	±	11.93	1	231.13	±	12.56	239.9	±	14.91	1

Values as mean and SD. P values calculated with ANOVA.

* P<0.05 compared to control group ≥17 yr.

$ P<0.05 compared to control group < 17 yr.

Volumetric bone mineral density (vBMD); bone mineral apparent density (BMAD).

**Table 8:** Adjusted Bone mineral content (BMC) in control and cyclists group under 17 (<17 yr) and equal or over (≥17 yr).

	Controls							Cyclists							
	<17 yr			≥17 yr			*P* $	<17 yr			≥17 yr			*P* *	*P* £
*BMC (g)*	Mean		SD	Mean		SD		Mean		SD	Mean		SD		
Whole body	2413.9	±	51.7	2474.8	±	64.5	0.781	2288.8	±	59.9	2283.8	±	54.8	0.171	0.565
Pelvis	277.2	±	9.5	310.9	±	11.9	1	264.1	±	11.0	245.7	±	10.1	0.001	0.017
Hip	37.3	±	1.1	42.1	±	1.4	1	39.6	±	1.3	38.1	±	1.2	0.216	0.015
Trochanter	9.4	±	0.4	10.1	±	0.5	1	9.3	±	0.5	8.7	±	0.4	0.214	0.123
Inter-trochanter	23.1	±	0.9	26.7	±	1.1	0.515	25.5	±	1.0	24.8	±	1.0	1	0.033
Femoral neck	4.9	±	0.2	5.3	±	0.2	1	4.8	±	0.2	4.6	±	0.2	0.055	0.084
Lumbar spine	58.0	±	2.6	65.3	±	3.3	1	56.9	±	3.1	56.7	±	2.8	0.312	0.199
Average arms	155.6	±	4.2	154.6	±	5.2	1	147.0	±	4.8	156.8	±	4.4	1	0.248
Average legs	506.4	±	11.0	503.7	±	13.8	0.055	459.1	±	12.8	440.5	±	11.7	0.007	0.516
Head	458.8	±	20.3	477.9	±	25.4	1	468.8	±	23.6	510.8	±	21.6	1	0.611

Values as mean and SD. P values calculated with repeated measures ANCOVA adjusted with total lean mass and height.

*P<0.05 compared to control group ≥17 yr.

$ P<0.05 compared to control group <17 yr.

£ P<0.05 interaction group × age.

**Table 9:** Adjusted Bone mineral density (BMD) and volumetric density (vBMD) in control and cyclists group under 17 (<17 yr) and equal or over (≥17 yr).

	Controls							Cyclists							
	<17 yr			≥17 yr			*P* $	<17 yr			≥17 yr			*P* *	*P* £
*BMD (g cm^−2^)*	Mean		SD	Mean		SD		Mean		SD	Mean		SD		
Whole body	1.113	±	0.025	1.164	±	0.025	1	1.075	±	0.023	1.099	±	0.021	0.316	0.543
Pelvis	1.151	±	0.031	1.27	±	0.039	0.489	1.063	±	0.036	1.02	±	0.033	0.001	0.023
Hip	1.016	±	0.023	1.085	±	0.029	1	1	±	0.027	0.954	±	0.024	0.008	0.03
Trochanter	0.843	±	0.21	0.874	±	0.026	0.650	0.789	±	0.024	0.737	±	0.22	0.001	0.076
Inter-trochanter	1.138	±	0.029	1.216	±	0.037	1	1.124	±	0.034	1.085	±	0.031	0.052	0.076
Femoral neck	0.918	±	0.025	1.009	±	0.031	1	0.934	±	0.029	0.874	±	0.026	0.01	0.008
Lumbar spine	0.928	±	0.025	1.012	±	0.032	1	0.906	±	0.029	0.916	±	0.027	0.148	0.191
Average arms	0.743	±	0.012	0.742	±	0.015	1	0.725	±	0.014	0.749	±	0.013	1	0.356
Average legs	1.269	±	0.024	1.318	±	0.03	0.182	1.186	±	0.027	1.21	±	0.025	0.045	0.633
Head	1.908	±	0.077	2.012	±	0.097	1	1.967	±	0.09	2.123	±	0.082	1	0.759
*vBMD (g cm^−3^)*															
Femoral neck	0.331	±	0.009	0.369	±	0.011	1	0.348	±	0.01	0.315	±	0.009	0.004	0.001
Lumbar spine	0.263	±	0.007	0.278	±	0.009	1	0.255	±	0.008	0.27	±	0.007	1	0.992
BMAD	0.089	±	0.035	0.186	±	0.043	1	0.092	±	0.04	0.093	±	0.037	0.634	0.211

Values as mean and SD. P values calculated with repeated measures ANCOVA adjusted with total lean mass and height.

*P<0.05 compared to control group ≥17 yr.

$ P<0.05 compared to control group <17 yr.

£ P<0.05 interaction group × age. Bone mineral apparent density (BMAD).

**Table 10:** Adjusted Bone mineral area in control and cyclists group under 17 (<17 yr) and equal or over (≥17 yr).

	Controls							Cyclists							
	<17 yr			≥17 yr			*P* $	<17 yr			≥17 yr			*P* *	*P* £
*Area (cm^−2^)*	Mean		SD	Mean		SD		Mean		SD	Mean		SD		
Whole body	2144.3	±	20.37	2113.46	±	25.42	0.695	2093.01	±	23.59	2067.12	±	21.58	1	0.543
Pelvis	236.15	±	5.23	241.71	±	6.53	1	242.5	±	6.06	240.12	±	5.55	1	0.912
Hip	36.39	±	0.68	38.55	±	0.84	0.053	39.31	±	0.78	39.78	±	0.72	1	0.262
Trochanter	11.03	±	0.28	11.4	±	0.35	1	11.59	±	0.32	11.72	±	0.29	1	0.695
Inter-trochanter	20.03	±	0.68	21.87	±	0.84	0.123	22.59	±	0.78	22.8	±	0.72	1	0.282
Femoral neck	5.32	±	0.08	5.28	±	0.1	0.797	5.13	±	0.1	5.25	±	0.09	1	0.357
Lumbar spine	61.43	±	1.48	63.97	±	1.85	1	62.22	±	1.71	61.59	±	1.57	1	0.335
Average arms	206.2	±	4.28	207.67	±	5.35	1	197.99	±	4.96	208.28	±	4.54	1	0.335
Average legs	396.16	±	6.16	380.55	±	7.69	0.641	380.23	±	7.14	362.23	±	6.53	0.448	0.86
Head	238.99	±	2.86	236.93	±	3.58	1	237.65	±	3.32	238.93	±	3.04	1	0.599

Values as mean and SD. P values calculated with repeated measures ANCOVA adjusted with total lean mass and height.

*P<0.05 compared to control group ≥17 yr.

$ P<0.05 compared to control group <17 yr.

£ P<0.05 interaction group × age.

Analyses were repeated including fat mass as covariable with no variation of the presented results.

**Figure 1:**
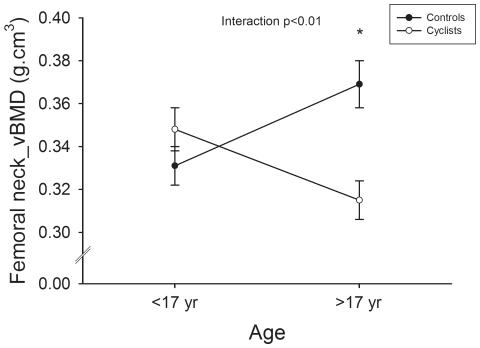
Group × age interaction for femoral neck volumetric bone mineral density (vBMD; P<0.01) in young and old adolescent cyclists and controls. * P<0.05 between control and cyclists groups ≥17 years.

Group interaction by age was found for BMC at the pelvis, hip and inter-trochanter sub-regions, and for BMD at the pelvis, hip and femoral neck (all P≤0.05, Table 8 & 9), as well as for vBMD at the femoral neck (P≤0.001, Figure 1).

All the previous comparisons exhibited large effect sizes (Cohen's d ranged from 0.8 to 7), excepting the hip BMD which were low (Cohen's d = 0.3).

## Discussion

The main finding herein is that adolescent cyclists had lower BMC and BMD compared with healthy age-matched controls in regions of clinical interest (hip, pelvis and femoral neck). Our study also shows that differences in BMC and BMD between cyclists and controls were higher in adolescents over 17 years old.

The present study shows that adolescent cyclists had lower BMC and BMD than healthy age-matched controls. Cross-sectional and longitudinal studies have shown that weight-bearing and impact-loading sports improve bone mass, especially at weight-bearing sites [22], [23], [24]. However, athletes who perform activities in which the body weight load is diminished or without impacts, as in cycling, are associated with a lower bone mass compared with athletes who participated in weight-bearing sports [25], [26]. Male professional cyclists had lower BMD for the whole body (12%), legs (16%), pelvis (18%), femoral neck (25%) [22] and lumbar spine [28] than non-active controls of similar age.

The differences observed in BMC and BMD in our adolescent cyclists are similar to those observed previously in professional cyclists who trained 3 times as much [22]. Sanchis et al. [27] found that young tennis players had 69% of the inter-arm asymmetry in BMC observed in professional tennis players who trained nearly twice as much, and all the asymmetry in bone area. In the review literature, we have found only 3 studies evaluating the bone mass in adolescent cyclists [11], [14], [15]. Rico et al. [14] did not find differences in total or regional BMC between male adolescent cyclists with a similar training frequency than in the present study (10 h/week), and age-matched controls, when values were corrected by body weight; one possible explanation that may explain this discrepancy is that in our study we corrected the BMC and BMD by the total lean mass and the height, as they are the variables having the highest effect on bone growth [4]. Unfortunately Rico et al. [14] did not evaluate BMD.

Duncan at al. [11] observed that female cyclists had similar BMD at whole body, lumbar, femoral neck, legs and arms than non-active population, but lower BMD at whole body, femoral neck and legs than a group of female runners. The same researchers compared total and cortical vBMD at the femur bone in adolescent females from different sport disciplines (cyclists, triathletes, swimmers and runners) and a non-active control group of the same gender [15]. Duncan et al (2002) showed that BMC and vBMD in mid femur was similar in all groups, except for runners who showed higher BMD values and bone strength than cyclists [15]. Several aspects may cause the differences between this latter research and the present one, such as different control groups (sedentary vs. actives) [28], differences in lean mass [24], or the known gender dimorphism in the bone development [29].

Our results showed that differences in BMC and BMD between cyclists and active controls were greater in adolescents over 17 years old than in those under that age. We also found a negative association between age and BMC, and BMD, in the cyclists. Unfortunately we only can compare our results with longitudinal studies conducted in adults. Nichols et al. [29] described the tracked changes in BMD over a 7-year period in competitive male master cyclists and non-athletes. Their results showed that at the beginning of the study, cyclists had lower lumbar and hip BMD than the control group; interestingly at the end of the study master cyclists had lost more BMD than controls [32]. A previous study examined BMD over a one year season in amateur male cyclists and found 1–1.5% decrease in BMD at the proximal femur but no changes at the lumbar spine [33]. Nichols et al. [8] observed that master cyclists (>50 yr) had lower total, lumbar and hip BMD than younger cyclists (mean 31 yr).

Bone mineral density is the main variable used to determine osteoporosis [30]. There is a close relationship between BMD and bone mechanical strength [31]. Our study shows that adolescent cyclists developed lower BMD than controls at relevant clinical sites. This could increase the risk of bone fractures and/or osteoporosis. However, in spite of lower levels of BMD at clinical sites, adult cyclists develop a higher cortical thickness which can also increase bone strength [32].

A recent study showed that master professional cyclists (>50 yr) exhibited greater BMC and cortical area at the tibiae and radius which was associated with higher polar momentum of resistance [32]. Longitudinal studies should be conducted to corroborate this finding and to analyze whether this effect can be generalized to include other bones of greater clinical interest [33].

Some limitations should be recognized. One is the design, from which it cannot be concluded that the effect that is observed in older adolescents is due to the longer period (years) of practice of cycling rather than internal (i.e. genetic) or external (i.e. energy imbalance) factors. The absence of hormone and calcium intake data is another potential weakness of this study because this may affect bone acquisition; although it could explain the mechanisms behind these observations they should not change the found lower bone mass found in cyclists.

Nevertheless, the analysis of the interaction between bone mass, age and cycling training may indicate that the practice of cycling training is linked to the lower bone mass found in our adolescents. In the same line we have found a strong negative correlation, after taking into account the age, height, muscle mass and years of practice, between hours of practice and BMC and BMD in all the regions studied in older adolescent cyclists (r = −0.31 to r = −0.76) although none of them reached statistical significance, maybe because the low sample. Nonetheless, this hypothesis must be corroborated with longitudinal or intervention studies.

A strength of this study is that volumetric density was calculated: vBMD and BMAD have been proposed as a better reflection of the real bone density [20], [34]. We detect no other study where these bone parameters were estimated in adolescent cyclists. In our study, we found a 17% lower vBMD at the femoral neck of the older cyclists compared to older controls, which may be associated with an important risk of fracture in this relevant clinical zone. The BMAD in the cyclist group was 100% lower than that in the controls in the older adolescents; although of no statistical significance, maybe because of the sample size, this may imply important biological consequences reflected by the high effect size.

### Conclusions

Our study shows that cycling training, may adversely affect bone mass during adolescence. Although this is a case control study and caution must be used in interpreting the results, the practice of cycling practice during adolescence may compromise the acquisition of bone mass.
